# Supplementary material for: Stress-activated MAPK signaling controls fission yeast actomyosin ring integrity by modulating formin For3 levels
Source: eLife. 2020 Sep 11;9:e57951. doi: 10.7554/eLife.57951 (PMC7511234; doi:10.7554/eLife.57951)
Supplement: Supplementary file 1. [file elife-57951-supp1.docx]

Supplementary file 1. *S. pombe* and *S. japonicus* strains used in this study

|  | **Figure 1** |  |
| --- | --- | --- |
| **Strain** | **Genotype** | **Source/Reference** |
| MM1 | h^+^ *ade6-M216 ura4D-18 leu1-32* | Madrid *et al.*(2006) |
| MI200 | h^+^ *ade6-M216 pmk1-HA6H:ura4^+^ ura4D-18 leu1-32* | Madrid *et al.* (2006) |
| MI204 | h^+^ *ade6-M216 sty1::ura4^+^  pmk1-HA:ura4^+^  ura4D-18 leu1-32* | Madrid *et al.* (2006) |
| MI709 | h^−^ *wis1DD-12myc::ura4*^+^ *pmk1-HA6H::ura4*^+^ *his7-336 ura4-D18 leu 1-32* | Madrid *et al.* (2007) |
| MI213 | h^+^ *ade6-M216 pyp1::kanR pmk1-HA6H:ura4^+^ ura4D-18 leu1-32* | Madrid *et al.* (2007) |
| MI220 | h^+^ *ade6-M216 pyp1::kanR sty1::ura4^+^ pmk1-HA6H:ura4^+^ ura4D-18 leu1-32* | Madrid *et al.* (2007) |
| MI216 | h^+^ *ade6-M216 ptc1::KanR pmk1-HA6H::ura4*^+^ *ura4D-18 leu1-32* | Madrid *et al.* (2007) |
| MI222 | h^+^ *ade6-M216 ptc1::KanR sty1::ura4*^+^ *pmk1-HA6H::ura4*^+^ *ura4D-18 leu1-32* | Madrid *et al.* (2007) |
| JM1521 | h^+^ *ade6-M216 sty1-HA6H:ura4^+^ his7-336 ura4D-18 leu1-32* | J.B.A. Millar |
| MW1541 | h^-^ *ade6-M216 mcs4::his7^+^ sty1-HA6H:ura4^+^  ura4D-18 leu1-32* | J.B.A. Millar |
| VB1700 | h^-^ *ade6-M216 mcs4^+^(D412N) sty1-HA6H:ura4^+^ his7-336 ura4D-18 leu1-32* | Buck *et a*l. (2001) |
| E288 | h^+^ *ade6-M216 sty1::natR ura4D-18 leu1-32* | This work |
| MY6940 | h^-^ *ade6-M216 atf1::ura4^+^ ura4D-18 leu1-32* | M. Yanagida |
| CA5931 | h^+^ *CRIB-3xGFP:ura4^+^ ade6-M216 ura4D-18 leu1-32* | K. Shiozaki |
|  |  |  |
|  | **Figure 2** |  |
| **Strain** | **Genotype** | **Source/Reference** |
| PPG135.29 | h^-^ *ade6-M216 pAct-Lifeact-GFP:leu1^+^ ura4D-18 leu1-32* | P. Pérez |
| E188 | h^?^  *ade6-M216 sty1::kanR pAct-Lifeact-GFP:leu1^+^ ura4D-18 leu1-32* | This work |
| E222 | h^?^ *ade6-M216 wis1DD-12myc::ura4*^+^ *pAct-Lifeact-GFP:leu1^+^ ura4D-18 leu1-32* | This work |
| E430 | h^+^ *ade6-M216 rlc1-GFP:kanR pcp1-GFP:kanR ura4D-18 leu1-32* | This work |
| E433 | h^?^ *ade6-M216 wis1DD-12myc::ura4*^+^ *rlc1-GFP:kanR pcp1-GFP:kanR ura4D-18 leu1-32* | This work |
| E1220 | h^?^ *ade6-M216 sty1::natR rlc1-GFP:kanR pcp1-GFP:kanR ura4D-18 leu1-32* | This work |
| PPG26.20 | h^-^ *ade6-M216 rlc1-GFP:kanR ura4D-18 leu1-32* | P. Pérez |
| E225 | h^?^ *ade6-M216 wis1DD-12myc::ura4*^+^ *rlc1-GFP:kanR ura4D-18 leu1-32* |  |
| E227 | h^?^ *ade6-M216 sty1::ura4^+^ rlc1-GFP:kanR ura4D-18 leu1-32* | This work |
| E963 | h^?^ *ade6-M216 sty1(T97A)-HA:ura4^+^ sty1::kanR rlc1-GFP:kanR ura4.294 leu1-32* | This work |
|  |  |  |
|  | **Figure 3** |  |
| **Strain** | **Genotype** | **Source/Reference** |
| MM1 | h^+^ *ade6-M216 ura4D-18 leu1-32* | Madrid *et al.*(2006) |
| E288 | h^+^ *ade6-M216 sty1::natR ura4D-18 leu1-32* | This work |
| PPG625.68 | h^+^ *ade6-M216 for3::kanR ura4D-18 leu1-32* | P. Pérez |
| E815 | h^?^  *ade6-M216 for3::kanR sty1::natR ura4D-18 leu1-32* | This work |
| E1213 | h^?^  *ade6-M216 for3::kanR rlc1-GFP:kanR pcp1-GFP:kanR ura4D-18 leu1-32* | This work |
|  |  |  |
|  | **Figure 4** |  |
| **Strain** | **Genotype** | **Source/Reference** |
| PPG56.71 | h^+^ *ade6-M216 for3-3xGFP:ura4^+^ ura4D-18 leu1-32* | P. Pérez |
| TK107 | h^-^ *ade6-M216 sty1::ura4^+^ ura4D-18 leu1-32* | T. Kato |
| PPG77.60 | h^+^ *ade6-M216 for3DAD*-2GFP:kanR ura4D-18 leu1-32* | Rincón *et al*. (2009) |
| E940 | h^?^  *ade6-M216 sty1::ura4^+^  for3DAD*-2GFP:kanR ura4D-18 leu1-32* | This work |
| E1286 | h^?^  *ade6-M216 for3-3xGFP:ura4^+^ rlc1-mCherry:kanR pcp1-GFP:kanR ura4D-18 leu1-32* | This work |
| E1288 | h^?^  *ade6-M216 for3DAD*-2GFP:kanR rlc1-mCherry:kanR pcp1-GFP:kanR ura4D-18 leu1-32* | This work |
| E1325 | h^?^  *ade6-M216 wis1DD-12myc::ura4*^+^ *for3-3xGFP:ura4^+^ rlc1-mCherry:kanR pcp1-GFP:kanR ura4D-18 leu1-32* | This work |
| E1328 | h^?^  *ade6-M216 wis1DD-12myc::ura4*^+^ *for3DAD*-2GFP:kanR rlc1-mCherry:kanR pcp1-GFP:kanR ura4D-18 leu1-32* | This work |
|  |  |  |
|  | **Figure 5** |  |
| **Strain** | **Genotype** | **Source/Reference** |
| PPG56.71 | h^+^ *ade6-M216 for3-3xGFP:ura4^+^ ura4D-18 leu1-32* | P. Pérez |
| E880 | h^?^ *ade6-M216 sty1::kanR for3-3xGFP:ura4^+^ ura4D-18 leu1-32* | This work |
| E1386 | h^?^ *ade6-M216 pyp1::natR for3-3xGFP:ura4^+^ ura4D-18 leu1-32* | This work |
| E884 | h^?^ *ade6-M216 wis1DD-12myc::ura4*^+^ *for3-3xGFP:ura4^+^ ura4D-18 leu1-32* | This work |
| E1058 | *h^?^ ade6-M216 sty1(T97A)-HA:ura4^+^ sty1::kanR for3-3xGFP:ura4^+^ ura4.294 leu1-32* | This work |
| E976 | h^?^  *ade6-M216 hht-RFP:kanR for3-3xGFP:ura4^+^ ura4D-18 leu1-32* | This work |
| E888 | h^?^ *ade6-M216 sty1-HA6H:ura4^+^ for3-3xGFP:ura4^+^ ura4D-18 leu1-32* | This work |
| E1726 | h^?^ *ade6-M216 cdc2.asM17:bsd for3-3xGFP:ura4^+^ ura4D-18 leu1-32* | This work |
| E1745 | h^?^ *ade6-M216 act1-LR:ura4^+^ for3-3xGFP:ura4^+^ ura4D-18 leu1-32* | This work |
| E336 | h^?^ *ade6-M216 wis1DD-12myc::ura4*^+^ *sty1-HA6H:ura4^+^ ura4D-18 leu1-32* | This work |
| MI1001 | h^+^ *ade6-M216 his7-336 pyp1::kanR sty1-HA6H:ura4^+^ ura4D-18 leu1-32* | Madrid *et al.* (2007) |
|  |  |  |
|  | **Figure 6** |  |
| **Strain** | **Genotype** | **Source/Reference** |
| PPG56.71 | h^+^ *ade6-M216 for3-3xGFP:ura4^+^ ura4D-18 leu1-32* | P. Pérez |
| E1612 | h^+^ *ade6-M216 sty1::natR for3-3xGFP:ura4^+^ ura4D-18 leu1-32* | P. Pérez |
| BV2915 | h^?^ *ade6-M216 cdc3-124 for3-3xGFP:ura4^+^ ura4D-18 leu1-32* | This work |
| BV3045 | h^?^ *ade6-M216 sty1::natR cdc3-124 for3-3xGFP:ura4^+^ ura4D-18 leu1-32* | This work |
| BV3000 | h^?^ *ade6-M216 cdc8-110 for3-3xGFP:ura4^+^ ura4D-18 leu1-32* | This work |
| BV3043 | h^?^ *ade6-M216 sty1::natR cdc8-110 for3-3xGFP:ura4^+^ ura4D-18 leu1-32* | This work |
|  |  |  |
|  | **Figure 7** |  |
| **Strain** | **Genotype** | **Source/Reference** |
| E1851 | h^?^ *ade6-M216 for3::kanR for3pr-for3-HA:ura4^+^ ura4.294 leu1-32* | This work |
| E1764 | h^?^  *ade6-M216 for3::kanR adh1-Z_3_EV:leu1^+^ Z_3_EVpr-For3-HA:ura4^+^ ura4.294 leu1-32* | This work |
| E1813 | h^?^  *ade6-M216 for3::natR adh1-Z_3_EV:leu1^+^ Z_3_EVpr-For3-HA:ura4^+^ rlc1-GFP:kanR pcp1-GFP:kanR ura4.294 leu1-32* | This work |
|  |  |  |
|  | **Figure 8** |  |
| **Strain** | **Genotype** | **Source/Reference** |
| NIG2028 | h^-^ | Furuya& Niki (2009) |
| TSJ101 | h^-^ *sty1::ura4^+^ ura4-D3* | Gómez-Gil *et al.* (2019) |
| TSJ105 | h^-^ *atf1::ura4^+^ ura4-D3* | Gómez-Gil *et al.* (2019) |
| SOJ1445 | h^?^  *ade6sj-domE? rlc1-GFP::kanR pcp1-mCherry::ura4+::kanR ura4sj-D3* | Gu, Yam &Oliferenko (2015) |
| TSJ 210 | h^?^ *ade6sj-domE? sty1::natR rlc1-GFP::kanR pcp1-mCherry::ura4+::kanR ura4sj-D3* | This work |
|  |  |  |
|  | **Figure 1 (supplements)** |  |
| **Strain** | **Genotype** | **Source/Reference** |
| JM1521 | h^+^ *ade6-M216 sty1-HA6H:ura4^+^ his7-336 ura4D-18 leu1-32* | J.B.A. Millar |
| MW1541 | h^-^ *ade6-M216 mcs4::his7^+^ sty1-HA6H:ura4^+^  ura4D-18 leu1-32* | J.B.A. Millar |
| TS-2 | h^-^ *ade6-M216 wak1::ura4^+^ sty1-HA6H:ura4^+^ ura4D-18 leu1-32* | Soto *et al*. (2002) |
| MR204 | h^?^ *ade6-M216 win1-1 sty1-HA6H:ura4^+^ ura4D-18 leu1-32* | M.A. Rodríguez-Gabriel |
| TS-3 | h^-^ *ade6-M216 wis1::his7^+^ sty1-HA6H:ura4^+^ ura4D-18 leu1-32* | Soto *et al*. (2002) |
| TK107 | h^-^ *ade6-M216 sty1::ura4^+^ ura4D-18 leu1-32* | T. Kato |
| E423 | h^?^ *ade6-M216 act1-LR:ura4^+^ sty1-HA6H::ura4^+^ ura4D-18 leu1-32* | This work |
| BV4 | h^+^ *ade6-M216 sty1::kanR sty1.DP(TGY)-HA6H:ura4^+^ ura4-294 leu1-32* | Vázquez *et al*. (2015) |
| BV6 | h^+^ *ade6-M216 sty1::kanR sty1.PT(TGF)-HA6H:ura4^+^ ura4-294 leu1-32* | Vázquez *et al*. (2015) |
| BV5 | h^+^ *ade6-M216 sty1::kanR sty1.PY (AGY)-HA6H:ura4^+^ ura4-294 leu1-32* | Vázquez *et al*. (2015) |
| BV7 | h^+^ *ade6-M216 sty1::kanR sty1.NP(AGF)-HA6H:ura4^+^ ura4-294 leu1-32* | Vázquez *et al*. (2015) |
| MM1 | h^+^ *ade6-M216 ura4D-18 leu1-32* | Madrid *et al.*(2006) |
| FPR177 | h*^-^ ade6-M216 sty1::ura4^+^ sty1-GFP:leu1^+^ ura4D-18 leu1-32* | Prieto *et al.* (2019) |
| E149 | h*^-^ ade6-M216 sty1::ura4^+^ sty1-GFP-CAAX:leu1^+^ ura4D-18 leu1-32* | This work |
| CA5931 | h^+^ *CRIB-3xGFP:ura4^+^ ade6-M216 ura4D-18 leu1-32* | K. Shiozaki |
| MM1 | h^+^ *ade6-M216 ura4D-18 leu1-32* | Madrid *et al.*(2006) |
| TK107 | h^-^ *ade6-M216 sty1::ura4^+^ ura4D-18 leu1-32* | T. Kato |
| PPG2517 | h^+^ *ade6-M216 gef1::kanR ura4D-18 leu1-32* | P. Pérez |
| E629 | h^?^ *ade6-M216 sty1::ura4^+^ gef1::kanR ura4D-18 leu1-32* | This work |
| E288 | h^+^  *ade6-M216 sty1::natR ura4D-18 leu1-32* | This work |
| PPG47.03 | h^+^  *ade6-M216 scd1::kanR ura4D-18 leu1-32* | P. Pérez |
| E1735 | h^?^ *ade6-M216 sty1::natR scd1::kanR ura4D-18 leu1-32* | This work |
|  |  |  |
|  | **Figure 2 (supplements)** |  |
| **Strain** | **Genotype** | **Source/Reference** |
| MM1 | h^+^ *ade6-M216 ura4D-18 leu1-32* | Madrid *et al.*(2006) |
| TK107 | h^-^ *ade6-M216 sty1::ura4^+^ ura4D-18 leu1-32* | T. Kato |
| 2119 | h^-^ *ade6-M216 wis1DD:12-myc:ura4^+^  ura4D-18 leu1-32* | M.A. Rodríguez-Gabriel |
| PPG135.29 | h^-^ *ade6-M216 pAct-Lifeact-GFP:leu1^+^ ura4D-18 leu1-32* | P. Pérez |
| E188 | h^?^  *ade6-M216 sty1::kanR pAct-Lifeact-GFP:leu1^+^ ura4D-18 leu1-32* | This work |
| E430 | h^+^ *ade6-M216 rlc1-GFP:kanR pcp1-GFP:kanR ura4D-18 leu1-32* | This work |
| E433 | h^?^ *ade6-M216 wis1DD-12myc::ura4*^+^ *rlc1-GFP:kanR pcp1-GFP:kanR ura4D-18 leu1-32* | This work |
| E1220 | h^?^ *ade6-M216 sty1::natR rlc1-GFP:kanR pcp1-GFP:kanR ura4D-18 leu1-32* | This work |
| E1213 | h^?^  *ade6-M216 for3::kanR rlc1-GFP:kanR pcp1-GFP:kanR ura4D-18 leu1-32* | This work |
|  |  |  |
|  | **Figure 3 (supplements)** |  |
| **Strain** | **Genotype** | **Source/Reference** |
| E320 | h^+^  *ade6-M216 cdc12-3HA:kanR ura4D-18 leu1-32* | This work |
| E376 | h^?^ *ade6-M216 sty1::ura4^+^ cdc12-3HA:kanR ura4D-18 leu1-32* | This work |
| E379 | h^?^ *ade6-M216 wis1DD-12myc::ura4*^+^ *cdc12-3HA:kanR ura4D-18 leu1-32* | This work |
| MM1 | h^+^ *ade6-M216 ura4D-1 8 leu1-32* | Madrid *et al.*(2006) |
| TK107 | h^-^ *ade6-M216 sty1::ura4^+^ ura4D-18 leu1-32* | T. Kato |
| KG13137 | h^-^ *ade6-M216 cdc12-4A:kanR ura4D-18 leu1-32* | Bohnert *et al*. (2013) |
| E712 | h^?^ *ade6-M216 sty1::ura4^+^ cdc12-4A:kanR ura4D-18 leu1-32* | This work |
| KG12700 | h^-^ *ade6-M216 cdc12-6D:kanR ura4D-18 leu1-32* | Willet *et al.* (2018) |
| E1403 | h^?^ *ade6-M216 sty1::ura4^+^ cdc12-6D:kanR ura4D-18 leu1-32* | This work |
| KG15568 | h^?^ *ade6-M216 cdc12-3xGFP:kan Rrlc1-mCherry:kanR ura4D-18 leu1-32* | Bohnert *et al*. (2013) |
| E1711 | h^?^ *ade6-M216 sty1::natR cdc12-3xGFP:kan Rrlc1-mCherry:kanR ura4D-18 leu1-32* | This work |
| KG15569 | h^?^ *ade6-M216 cdc12-4A-3xGFP:kanR:hygR rlc1-mCherry:kanR ura4D-18 leu1-32* | Bohnert *et al*. (2013) |
| E1712 | h^?^ *ade6-M216 sty1::natR cdc12-4A-3xGFP:kanR:hygR rlc1-mCherry:kanR ura4D-18 leu1-32* | This work |
|  |  |  |
|  | **Figure 5 (supplements)** |  |
| **Strain** | **Genotype** | **Source/Reference** |
| PPG56.71 | h^+^ *ade6-M216 for3-3xGFP:ura4^+^ ura4D-18 leu1-32* | P. Pérez |
| E1002 | h^?^  *ade6-M216 cdc25-22 for3-3xGFP:ura4^+^ ura4D-18 leu1-32* | This work |
| E969 | h^?^ *ade6-M216 cdc10-129 for3-3xGFP:ura4^+^ ura4D-18 leu1-32* | This work |
| E932 | h^?^ *ade6-M216 nda3-K311 for3-3xGFP:ura4^+^ ura4D-18 leu1-32* | This work |
| E880 | h^?^ *ade6-M216 sty1::kanR for3-3xGFP:ura4^+^ ura4D-18 leu1-32* | This work |
| E884 | h^?^  *ade6-M216 wis1DD-12myc::ura4*^+^ *for3-3xGFP:ura4^+^ ura4D-18 leu1-32* | This work |
| E1570 | h^?^ *ade6-M216 atf1::kanR for3-3xGFP:ura4^+^ ura4D-18 leu1-32* | This work |
| E1838 | h^+^ *ade6-M216 pREP-polyHis-Ub:leu1^+^ ura4D-18 leu1-32* | This work |
| E1832 | h^+^ *ade6-M216 pREP-polyHis-Ub:leu1^+^ for3-3xGFP:ura4^+^ ura4D-18 leu1-32* | This work |
| E1876 | h^?^ *ade6-M216 pREP-polyHis-Ub:leu1^+^ for3-3xGFP:ura4^+^ mts3-1 ura4D-18 leu1-32* | This work |
