# Supplementary material for: Stress-activated MAPK signaling controls fission yeast actomyosin ring integrity by modulating formin For3 levels
Source: eLife. 2020 Sep 11;9:e57951. doi: 10.7554/eLife.57951 (PMC7511234; doi:10.7554/eLife.57951)
Supplement: Supplementary file 2. [file elife-57951-supp2.docx]

| **OLIGONUCLEOTIDE** | **SEQUENCE 5’-3’** | **Use** |
| --- | --- | --- |
| Sty1DEL-F | TATAGACGAAGGACGCTTAAATTTTTGAGATTATTGTTGAATAGTCCTTTTTGTAACCAGTTTGAATAAA CGGATCCCCGGGTTAATTAA | *sty1^+^ deletion* |
| Sty1DEL-R | ACACGTGAACAAAATAGAGTAATCATAACATACCCCGAGAACAACTTTTAAGGCTTTATCTACAACTTGTGAATTCGAGCTCGTTTAAAC | *sty1^+^ deletion* |
| Sty1COM-F | CAC AAC TTA ACC AGA GCA CTT CAT T | *Confirmation of sty1^+^ deletion* |
| Pcp1-CTAG-F | GTG GCT AAA ACA AGC TCA ATT GAA ACA ATC ATT GCA AAG AGC TGC CGC AAA GGC AAA GACCGC AAA CTA CCG GAT CCC CGG GTT AAT TAA | *pcp1^+^ C-terminal tagging* |
| Pcp1-CTAG-R | ATA ATT ATA GTA GTA GAA TTA ATT GAA TGT TGT TAA AAA AAA AGA GAG TAA AAA ACG TAA GTA TCC CAG AGA ATT CGA GCT CGT TTA AAC | *pcp1^+^ C-terminal tagging* |
| Sty1-AS(T97A)-F | GATATTTATTTTGTCGCGGAGCTTCTGGGAACA | *Sty1 threonine-97 replaced by alanine (site-directed mutagenesis)* |
| Sty1-AS(T97A)-R | TGTTCCCAGAAGCTCCGCGACAAAATAAATATC | *Sty1 threonine-97 replaced by alanine (site-directed mutagenesis)* |
| Sty1SEQ1-R | GGT CTT GAA TAC GAG CCA AA | *sty1^+^ sequencing* |
| Sty1SEQ2-F | GCG ATT TAA AAA TCT GCG ATT TCG G | *sty1^+^ sequencing* |
| For3-DEL-F | TTC CTT ACC ATT TAT TCC TTA ATC AGC TTC GTT AGT ATC TTT TTT ACA ACC AAA TTA CCA GTT TGGTAT GTT AAT TCA TAC GGA TCC CCG GGT TAA TTA A | *for3^+^ deletion* |
| For3-DEL-R | TCT TTC AGA CAA ATC GTC AAT GTA TGT AAT GTA CAG ATA TAC TGT TCT AAA AAT CCA TCC TAGAAA GAA CAA TGG AGC AAG AAT TCG AGC TCG TTT AAA C | *for3^+^ deletion* |
| For3-COM-F | GCT GGT GTT GAG GTT GCT ATA TAA TA | *Confirmation of for3^+^ deletion* |
| Pyp1DEL-F | TTT AAT CAT CTC TGC TTC TTT TTT AAG GCC AAA TAT TCT TAA TAC AAA ACA TTC ATC TAA AAA ACC ACG ACG GAT CCC CGG GTT AAT TAA | *pyp1^+^ deletion* |
| Pyp1DEL-R | CAA GTA CAA GAA ATA AAG GAA TCG ATT AAA ACA CGA ATA TAT ATT GCC AAG AAA AAT CCA GTC AAA AAT TGA ATT CGA GCT CGT TTA AAC | *pyp1^+^ deletion* |
| KAN-COMP-R | GATGTGAGAACTGTATCCTAGCAAG | *Common oligonucleotide for confirmation of gene tagging* |
| NAT-R | CTCATGTAGAGCGCCTGCCGC | *Common oligonucleotide for confirmation of gene tagging* |
| XhoI-Z3EVPR-F | TATCTCGAGAGGAAGTTATATTGAATTTTCAA | *Cloning of Z_3_EVpr sequence into pJK210* |
| SmaI-Z3EVPR-R | AATCCCGGGTATAGTTTTTTCTCCTTGAC | *Cloning of Z_3_EVpr sequence into pJK210* |
| For3-PJK210ZEV-F(SmaI) | TATATCCCGGGATGGCATCTAAAATGCCTGAAGGGT | *Cloning of HA tagged for3^+^ into Z_3_EVpr-pJK210* |
| For3-HA-PJK210ZEV-R(SacII) | TATATCCGCGGCTATGCATAGTCCGGGACGTCATAGGGATAGCCTTGTTTTTGGCGGTCATTTTCA | *Cloning of HA tagged for3^+^ into Z_3_EVpr-pJK210* |
| ProFor3-SalI-F | TATATGTCGACTTAAATGAATTTCAAATTAACAGAA | *Cloning of for3pr and HA tagged for3^+^ into pJK210* |
| For3-HA-PJK210ZEV`R (XmaI) | TATATCCCGGGCTATGCATAGTCCGGGACGTCATAGGGATAGCCTTGTTTTTGGCGGTCATTTTCA | *Cloning of for3pr and HA tagged for3^+^ into pJK210* |
| For3Pro-seq-R | GAGTAACTGGAATTTCGAGATTGAA | *for3 promoter sequencing* |
| For3-SEQ1-R | ACA GTA AAC TTT CAA TAC CA | *for3^+^ sequencing* |
| For3-SEQ2-F | ACG ATG ATG CTC CTC TTA CT | *for3^+^ sequencing* |
| For3-SEQ3-F | ATG TGA TTA CTG GTG TCC GA | *for3^+^ sequencing* |
| For3-SEQ4-F | GCA GAT ACT AAT GTC GGT CT | *for3^+^ sequencing* |
| For3-SEQ5-F | TAG TTC CAA CAC CAG CTC CA | *for3^+^ sequencing* |
| For3-SEQ6-F | TGC CTA TAC CGA TCC ATT CA | *for3^+^ sequencing* |
| For3-SEQ7-F | GCG TAC GAT ATT CTT TCC GA | *for3^+^ sequencing* |
| Cdc12-TAG-F | ACC ACA ACG AGC ACA AGA AAT GCT TGC AGG TTT ATT ATC TGG AAA ATT GGC GCC TAA GGAGAA TGA GAA ACG GAT CCC CGG GTT AAT TAA | *cdc12^+^ C-terminal tagging* |
| Cdc12-TAG-R | AAA ATG ATG AAC ATG ATA CAC ACT TCA AAA AAA GGG CAA TCT TCC AAC TAA ACT AAC TTCCAA AAT TAG AGA ATT CGA GCT CGT TTA AAC | *cdc12^+^ C-terminal tagging* |
| Cdc12-COM-F | ATT AGT ATC GCC TTC CGT TTC TAT T | *Confirmation of cdc12^+^ C-terminal tagging* |
| STY1-DJP-X (NAT) | TTAATTAACCCGGGGATCCGACACCAGCACTGTGGACGTACTTC | *sty1^+^* deletion (*NatR*) in *S. japonicus.* |
| STY1-DJP-Y (NAT) | GTTTAAACGAGCTCGAATTCGACGATCTGCAGCAAGAATACATTG | *sty1^+^* deletion (*NatR*) in *S. japonicus.* |
| STY1-DJP-W | GCATGGGCGCGTTCGGTCTTGTATG | *sty1^+^* deletion in *S. japonicus.* |
| STYDJP-Z | TGCTTGCACCTCACTATCCACATTATG | *sty1^+^* deletion in *S. japonicus.* |
| Sty1DJP-COMP5’ | ATGGCTGAATTTGTTCGTACACAGAT | Confirmation of *sty1^+^*deletion in *S. japonicus.* |
|  |  |  |
| Leu1-FWD | CTTCCCTTCTCCTTCGTTATGG | *q-PCR* |
| Leu2-REV | CCTCCCAAATCGCGAGTATAAA | *q-PCR* |
| For3-FWD | GTGAAATATCTTCCTCGCCTTC | *q-PCR* |
| For3-REV | CTGGAGGAGGAACTGGAATAG | *q-PCR* |
| Act1-FWD | GGACTCTGGTGATGGTGTTA | *q-PCR* |
| Act1-REV | TTCGGCGGTAGTAGAGAAAG | *q-PCR* |
|  |  |  |
| GST-For31-F(SmaI) | TTAATC CCG GGA ATG GCA TCT AAA ATG CCT GAA GGG | *Cloning of For3_1_ fragment (amino acids 1-390) into pGEX-KG.* |
| GST-For31-R(NcoI) | TTAAT CCA TGG TTA GTT TCT CCA TTT TAA CAA AGA GTC | *Cloning of For3_1_ fragment (amino acids 1-390) into pGEX-KG.* |
| GST-For32-F(SmaI) | TTAATC CCG GGA AGA CAC GTC CGT GAT GCT CTG CAA | *Cloning of For3_2_ fragment (amino acids 391-920) into pGEX-KG.* |
| GST-For32-R(NcoI) | TTAAT CCA TGG TTA TAA AAG AGA AGA TCG TTG GTC AGA | *Cloning of For3_2_ fragment (amino acids 391-920) into pGEX-KG.* |
| GST-For33-F(SmaI) | TTAATC CCG GGACCT GAT ACA GTT GAA CAA AAT ATG | *Cloning of For3_3_ fragment (amino acids 921-1461) into pGEX-KG.* |
| GST-For33-R(NcoI) | TTAAT CCA TGG CTATTGTTTTTGGCGGTCATTTTCAAC | *Cloning of For3_3_ fragment (amino acids 921-1461) into pGEX-KG.* |
